# Internal hernia beneath superior vesical artery after pelvic lymphadenectomy for cervical cancer: a case report and literature review

**DOI:** 10.1186/s12893-020-00985-4

**Published:** 2020-12-02

**Authors:** Wen Ai, Zhihua Liang, Feng Li, Haihua Yu

**Affiliations:** The First Affiliated Hospital of Shandong First Medical University, Jinan, 250014 Shandong China

**Keywords:** Internal hernia, Perforation, Superior vesical artery, Laparoscopic pelvic lymphadenectomy

## Abstract

**Background:**

The common complications of radical hysterectomy and pelvic lymphadenectomy usually include wound infection, hemorrhage or hematomas, lymphocele, uretheral injury, ileus and incisional hernias. However, internal hernia secondary to the orifice associated with the uncovered vessels after pelvic lymphadenectomy is very rare.

**Case presentation:**

We report a case of internal hernia with intestinal perforation beneath the superior vesical artery that occurred one month after laparoscopic pelvic lymphadenectomy for cervical cancer. A partial ileum resection was performed and the right superior vesical artery was transected to prevent recurrence of the internal hernia.

**Conclusions:**

Retroperitonealization after the pelvic lymphadenectomy should be considered in patients with tortuous, elongated arteries which could be causal lesions of an internal hernia.

## Background

Radical hysterectomy and lymphadenectomy is a standard procedure in the radical surgery for cervical cancer. The common complications of radical hysterectomy and pelvic lymphadenectomy usually include wound infection, hemorrhage or hematomas, lymphocele, uretheral injury, ileus and incisional hernias [1]. However, internal hernia secondary to the orifice associated with the skeletonized vessels is very rare. Here we first report a case of internal hernia beneath superior vesical artery after pelvic lymphadenectomy for cervical cancer and conduct a literature review.

## Case presentation

**Fig. 1:**
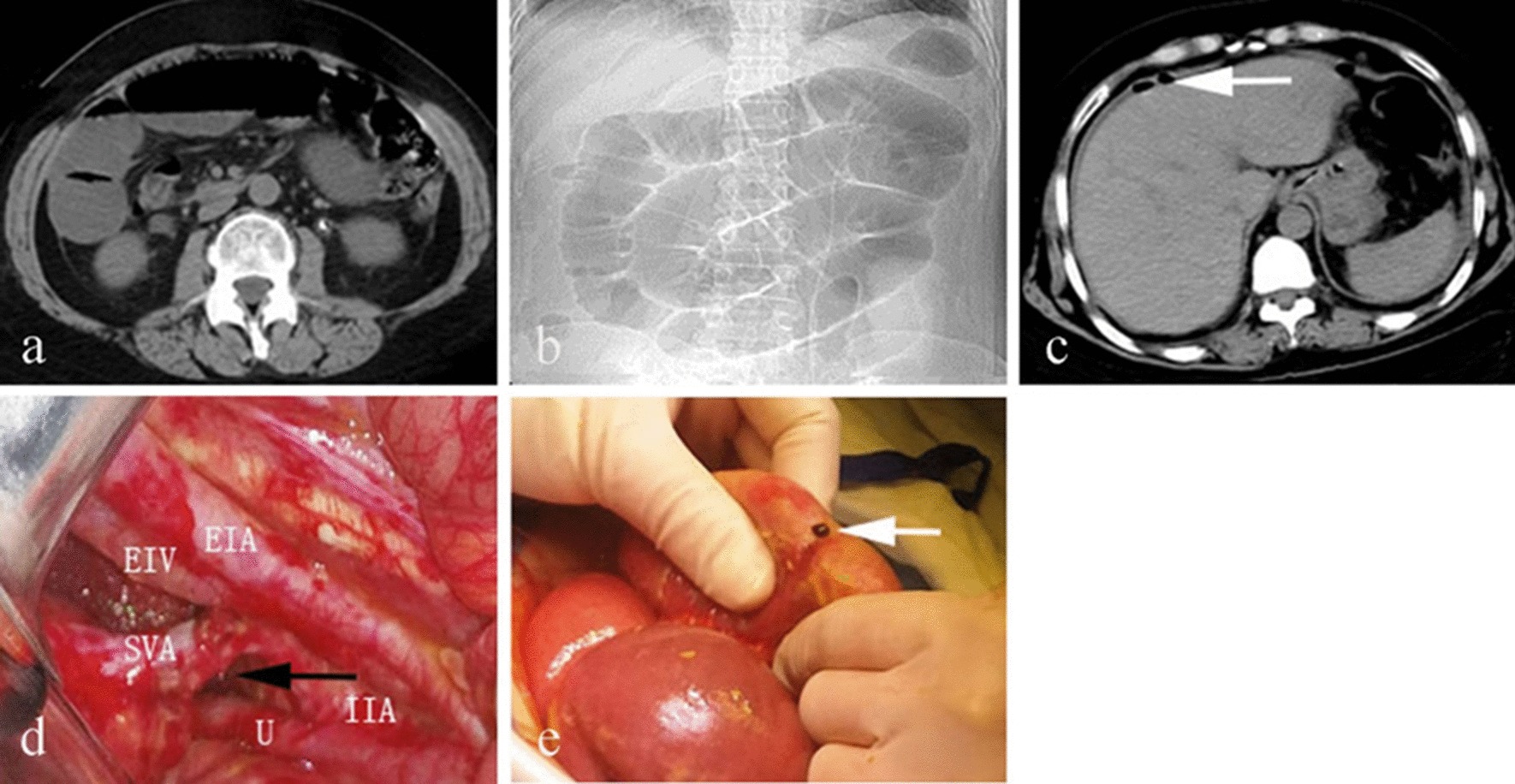
**a** CT scan revealed small intestine gas–liquid plane. **b** Small bowel obstruction. **c** White arrow for free gas in the abdominal cavity. **d** *EIA* external iliac artery. *EIV* external iliac vein. *IIA* internal iliac artery. *U* ureter. *SVA* superior vesical artery, black arrow for hernia ring defect. **e** White arrow shows the outflow of digestive juice

A 53-year-old woman underwent a laparoscopic radical hysterectomy, pelvic lymphadenectomy, para-aortic lymph node dissection and bilateral salpingo-oophorectomy for cervical cancer. In addition, a double J tube was placed in the left ureter for the sake of intraoperative urethral injury. The surgical pathology showed moderately differentiated squamous cell carcinoma and tumour metastasis was not found in the dissected 71 lymph nodes (Stage IB3). The postoperative hospital stay was uneventful and the patient was discharged 15 days after surgery. Two weeks later, she was admitted to our hospital again with a 5-day history of abdominal pain, vomiting, and the inability to pass gas or stools. Physical examination showed the abdominal distension, tenderness and hyperactive bowel sounds without rebound tenderness and muscular defense. Generally, the laboratory findings were not remarkable. CT scan revealed small bowel obstruction (Fig. 1a).

Based on these data, the patient was tentatively diagnosed as adhesive small bowel obstruction and then she received comprehensive conservative treatment. However, no significant improvement was observed in the ileus condition. On the sixth day, her abdominal pain suddenly aggravated with mild fever (37.3℃) and physical examination showed severe abdominal tenderness with rebound tenderness, especially in the hypogastric region. Laboratory data showed the elevation of white blood cell count (10.75 × 10^9^ /L, 3.5–9.5 × 10^9^ /L) and neutrophil granulocyte percentage (0.912, 0.40–0.75). CT scan revealed the ileus with a caliber change of the small bowel in the right lateral pelvic cavity and free gas in the abdominal cavity which suggested perforation of intestine (Fig. 1b, c). In view of the patient's acute abdominal condition, an emergency exploratory laparotomy was performed.

To avoid the possible adhesion of umbilicus from the previous operation and extend incision easily, we made a 12 cm incision through the rectus abdominis and found there were about 200 ml pus and digestive juices in the abdominal cavity. Intraoperative exploration revealed severe intestinal dilation and the incarcerated internal herniation of the distal ileum (20 cm length, 40 cm from Bauhin’s valve) through an orifice formed by the uncovered right superior vesical artery and the right lateral pelvic wall (Fig. 1d). After reduction of the herniated intestine, the incarcerated small bowel recovered good vitality. However, a 0.8 cm size perforation was found in the contralateral mesenteric region of the ileum about 80 cm far from Bauhin’s valve with leakage of digestive fluid (Fig. 1e). In addition, the intestinal wall surrounding the perforation was highly congested and a partial ileum resection was performed. Then the right superior vesical artery was transected to prevent recurrence of the internal hernia. The postoperative course was uneventful, and the patient was discharged from hospital on the tenth day after operation.

## Discussion and conclusions

Radical hysterectomy and pelvic lymphadenectomy is the recommended surgical option for stage IB1, IB2 and partial IB3 cervical cancer according to the NCCN guidelines [2]. Laparoscopic surgery has gradually been accepted as a safe and feasible procedure for cervical cancer in recent years. The intestinal obstruction, most of the cases due to adhesion, accounting for up to 75% of postoperative complications, meanwhile, 0.5–5.0% for internal hernia [3]. Moreover, internal hernia secondary to the orifice associated with the uncovered vessels after lymphadenectomy is very rare.

To the best of our knowledge, Guba et al. first reported an iatrogenic internal hernia beneath the right iliac artery after lymphadenectomy in testicular cancer in 1978 and up to till now, only eight cases were reported in English literatures. The previous reports are shown in Table 1 [4–11]. Except for one laparotomy [4], the remaining 7 documents (8 cases) were all laparoscopic or robotic surgery. Hiki et al*.* reported the adhesion after laparoscopic or robotic surgery was obviously better than that of open surgery [12]. According to the Japanese literature, 200 (laparotomy) versus 276 (laparoscopy) cases of lateral lymph node dissection, there are only 2 cases of internal hernia occurred and all of them are in the laparoscopy group [11]. Minami [10] conjectures the incidence of strangulated bowel obstruction may rise with the increasing number of laparoscopic or robot-assisted pelvic lymphadenectomies, although they have less postoperative adhesion formation than open surgery. However, due to lack of bulk data support, it is worthy of discussion whether internal hernia may increase in future on account of more and more laparoscopic lymphadenectomy surgeries.

**Table 1:** Previous reports of internal hernia after pelvic lymphadenectomy

Year/authors	Age/sex	Diagnosis	Operation method	Interval between surgery and hospital readmission	“Hernia ring” structure	Retroperitonization or not	Surgical treatment for the orifice
1978 Guba et al. [4]	52 M	Testicular teratoma	Laparotomy	4 months	Right common iliac artery	No	Free peritoneal transplantation
2008 Kim et al. [5]	67F	Cervical cancer	Laparoscopic	3 months	Right external iliac artery	No	Free peritoneal transplantation
2013 Dumant et al. [6]	56F	Ovarian cancer	Laparoscopic and laparotomy	4 years	Left external iliac artery	No	No special handling
2014 Ardelt et al. [7]	39F	Cervical cancer	Laparoscopic	2 years	Right common iliac artery	Not mentioned	Gluing a collagen patch
2015 Pridgian et al. [8]	50 M	Bladder cancer	Robotic	5 months	Right common iliac artery	Not mentioned	Free peritoneal transplantation
2016 Viktorin-Baier et al. [9]	50 M	Prostate cancer	Robotic	1 year	Left external iliac artery	Not mentioned	Securing by a fibrin sealant patch
2017 Minami et al. [10]	38F	Cervical cancer	Laparoscopic	6 months	Umbilical artery and obturator nerve	No	Resecting the umbilical artery
2018 Kitaguchi et al. [11]	68 M/59 M	Rectal cancer/cectal cancer	Laparoscopic/laparoscopic	4 months/2 months	superior vesical artery superior vesical artery	Not mentioned	No special handling/resecting the umbilical artery

The standard radical lymphadenectomy procedure for pelvic cancer usually includes the skeletonization of the pelvic nerves and iliac vessels from neighboring tissues which potentially create an iatrogenic hernia defect. Currently, retroperitonization after lymphadenectomy is rarely performed by gynecologists or urologists. Franchi reported there are no differences in postoperative complications between closure and no closure of the peritoneum [13]. Kadanali believed that unreconstructed peritoneum can reduce the incidence of adhesion [14]. At present, there has been no exact conclusion whether it is more beneficial to retroperitonize after pelvic lymphadenectomy. Measures dealing with hernia defects include omentum coverage, free peritoneum transplantation, partial vascular excision, mesh repair etc*.* and free peritoneum transplantation is most widely used due to its universality and accessibility [4, 5, 8]. In our case, the right superior vesical artery was transected.

When one patient presents with abdominal pain and intestinal obstruction after pelvic lymphadenectomy, it is important to distinguish the internal hernia from adhesive ileus, because the former is more life-threatening and mostly needs emergency surgery while the latter doesn’t. Although clinical symptoms can not effectively differentiate the two conditions because they are nonspecific, computed tomography (CT) is usually useful for this situation. The characteristic CT finding is the caliber change of the small bowel in the lateral pelvic cavity or the caudal dorsal side of pelvic vessels or nerves [11].

In summary, surgeons should be aware of the increased possibility of internal hernia in patients undergoing laparoscopic pelvic lymphadenectomy. Pelvic retroperitonealization after pelvic lymphadenectomy should be reconsidered for the prevention of internal hernia, especially for laparoscopic or robotic surgery.

## Data Availability

Not applicable.

## Abbreviations

CT: Computed tomography
EIA: External iliac artery
EIV: External iliac vein
IIA: Internal iliac artery
U: Ureter
SVA: Superior vesical artery
